# Urogenital pathogens, associated with *Trichomonas vaginalis*, among pregnant women in Kilifi, Kenya: a nested case-control study

**DOI:** 10.1186/s12879-018-3455-4

**Published:** 2018-11-06

**Authors:** Simon C. Masha, Piet Cools, Patrick Descheemaeker, Marijke Reynders, Eduard J. Sanders, Mario Vaneechoutte

**Affiliations:** 1 Kenya Medical Research Institute, Centre for Geographic Medicine Research – Coast, Kenya Medical Research Institute, P.O. Box 230, Kilifi, Kenya; 2 Laboratory Bacteriology Research, Faculty of Medicine and Health Sciences, Ghent University, De Pintelaan, 185 Ghent, Belgium; 3 Faculty of Pure and Applied Sciences, Department of Biological Sciences, Pwani University, Kilifi, Kenya; 4 Department of Laboratory Medicine, Medical Microbiology, AZ St-Jan Brugge-Oostende, Bruges, Belgium

**Keywords:** *Trichomonas*, *Mycoplasma hominis*, *M. Genitalium*, *M. Girerdii*, Kenya, Pregnant, STIs

## Abstract

**Background:**

Screening of curable sexually transmitted infections is frequently oriented towards the diagnosis of chlamydia, gonorrhea, syphilis and trichomoniasis, whereas other pathogens, sometimes associated with similar urogenital syndromes, remain undiagnosed and/or untreated. Some of these pathogens are associated with adverse pregnancy outcomes.

**Methods:**

In a nested case-control study, vaginal swabs from 79 pregnant women, i.e., 28 *T. vaginalis*-positive (cases) and 51 *T. vaginalis*-negative (controls), were screened by quantitative PCR for Adenovirus 1 and 2, Cytomegalovirus, Herpes Simplex Virus 1 and 2, *Chlamydia trachomatis, Escherichia coli, Haemophilus ducreyi, Mycoplasma genitalium, M. hominis,* candidatus *M. girerdii*, *Neisseria gonorrhoeae, Streptococcus agalactiae*, *Treponema pallidum, Ureaplasma parvum, U. urealyticum*, and *Candida albicans.* Additionally, we determined whether women with pathogens highly associated with *T. vaginalis* had distinct clinical signs and symptoms compared to women with *T. vaginalis* mono-infection.

**Results:**

*M. hominis* was independently associated with *T. vaginalis* (adjusted odds ratio = 6.8, 95% CI: 2.3–19.8). Moreover, *M. genitalium* and Ca *M. girerdii* were exclusively detected in women with *T. vaginalis* (*P* = 0.002 and *P* = 0.001), respectively. Four of the six women co-infected with *T. vaginalis* and Ca *M. girerdii* complained of vaginal itching, compared to only 4 out of the 22 women infected with *T. vaginalis* without Ca *M. girerdii* (*P* = 0.020).

**Conclusion:**

We confirm *M. hominis* as a correlate of *T. vaginalis* in our population, and the exclusive association of both *M. genitalium* and Ca. *M. girerdii* with *T. vaginalis*. Screening and treatment of these pathogens should be considered.

**Electronic supplementary material:**

The online version of this article (10.1186/s12879-018-3455-4) contains supplementary material, which is available to authorized users.

## Background

Sexually transmitted infections (STIs) constitute a huge proportion of the most prevalent acute infections globally [1]. The most prevalent curable sexually transmitted pathogens include *Chlamydia trachomatis*, *Neisseria gonorrhoeae*, *Treponema pallidum* subspecies *pallidum* (syphilis) and *Trichomonas vaginalis*. These four pathogens are associated with acute conditions like genital/anorectal/oral ulceration, cervicitis-endometritis, vaginal/urethral discharge, and urethritis. They can also cause critical complications and long term sequelae, which includes oophoritis, salpingitis, pelvic inflammatory disease, ectopic pregnancy, infertility, neurological disease, neonatal death, premature delivery and blindness [2]. Another public health concern is the association of STIs with the augmented possibility of HIV acquisition and transmission [3].

Of the four most prevalent curable STIs, *T. vaginalis* is globally the most prevalent pathogen [1], with a prevalence of up to 11.5% among women in sub-Sahara Africa [1]. Although there is a wealth of data regarding the clinical presentation and global burden of *T. vaginalis* [4], studies assessing associations of *T. vaginalis* with other genital pathogens are scarce, although it has been intimated that *T. vaginalis* has a unique symbiotic relationship with *Mycoplasma hominis* (*M. hominis*) [5]. *T. vaginalis* is also associated with an increase in vaginal pH [6] which may influence the composition of the associated vaginal microbial community and is strongly associated with bacterial vaginosis (BV) [6]. Less appreciated pathogens like *M. hominis*, *M. genitalium*, *Ureaplasma parvum* and *U. urealyticum* are increasingly being associated with adverse pregnancy outcomes as well as respiratory infections in neonates [7].

STI screening programs and STI research mostly focus on the four most common curable STIs, whereas other pathogens, associated with similar urogenital syndromes, remain undiagnosed and/or untreated. Detection of urogenital pathogens that may be associated with *T. vaginalis* may be important as it might have an effect on the clinical presentation and management and the long-term outcome of those infections. Here, we assess the occurrence of specific urogenital species in *T. vaginalis*-positive (cases) and *T. vaginalis*-negative (controls) among pregnant women in Kilifi, Kenya.

## Methods

### Study setting

Kenya, an East African country, is divided administratively into 47 counties. Kilifi County, which lies along the Indian Ocean Coast, is one of the poorest and is typical of a rural equatorial Africa setting. Among pregnant women in Kilifi the HIV prevalence is estimated to be 6.4%, for chlamydia 14.9%, for gonorrhea 1.0 and 7.4% for trichomoniasis [8].

From July till September 2015, we carried out a curable STI study at the prenatal care clinic of Kilifi County Hospital, Kenya. The key objective of the curable STI study was to illustrate the prevalence and predictors of curable sexually transmitted infections (STIs) among 350 pregnant women attending the prenatal care clinic [8]. The eligibility criteria for the curable STI study included: residing in the Kilifi Health and Demographic Surveillance area, age 18–45 years, willingness to undergo free STI and bacterial vaginosis (BV) screening procedures, gestation ≥14 weeks, and willing to give written informed consent. This study presents a secondary aim of the curable STI study, which is to describe urogenital pathogen correlates of *T. vaginalis* among pregnant women in Kilifi, Kenya.

For the above-described curable STI study, a nurse at the prenatal care clinic collected vaginal secretions from the vaginal introitus using two sterile cotton swabs. The first vaginal swab was used for *T. vaginalis* detection using the InPouch system (BioMed Diagnostics, White City, Oregon, USA), a highly specific and sensitive device containing a fluid medium supporting the growth of *T. vaginalis* and allowing microscopic observation of *T. vaginalis*. The inoculated InPouch was transported to the laboratory within 15 min for direct microscopy, and incubation at 37 °C ± 1 °C. Daily microscopic observation (at both × 10 and × 40 magnification, for six fields) of the InPouch system was performed by qualified technicians. Motile trichomonads within 5 days of culture were indicative of being positive for *T. vaginalis*.

Same day treatment was offered for women who were determined to be positive for *T. vaginalis* by means of direct microscopy. For women whose culture turned positive but were negative for *T. vaginalis* on direct microscopy, they were contacted to return to the clinic for treatment the moment the culture turned positive. Secnidazole 2 g statim was administered as treatment, and participants were also asked to refer their sexual partner(s) to the clinic for treatment or were given the same medication to take to their sexual partner(s).

The second swab had its shaft broken by bending the shaft against the neck of a sterile, labeled 2 ml Eppendorf tube, the tube containing the swab tip was closed and transferred to the laboratory where it was immediately stored at − 80 °C. No transport or freezing medium was added prior to storage.

Specimens for this case-control study are derived from the stored swabs from the curable STI study, published previously [7]. Because of financial and logistic constraints we could process only a subset of the 350 vaginal swabs from the main study. Vaginal swabs from 79 pregnant women were divided in two groups for analysis, i.e., those from women positive for *T. vaginalis* (cases) and those from women negative for *T. vaginalis* (controls) as determined by PCR. Controls were age-matched (+/− 5 years) and all were bacterial vaginosis (BV) negative by Nugent score, largely matching the cases because only 4 out of the 28 TV+ cases were BV+. Selection of controls was guided by being TV negative. The swabs of 51 women selected as controls were not significantly different from the swabs of the other 271 women not selected as controls (Additional file 1: Table S1).

### DNA extraction

Before DNA extraction, which was performed in Kilifi, the frozen swabs were thawed at room temperature (approximately 25 °C) for 30 min. Extraction was performed using the QIAamp DNA Mini Kit (Qiagen, Hilden, Germany) according to manufacturer’s instructions and 160 μl of eluted DNA was transferred to Eppendorf tubes and frozen at − 80 °C until shipment to the Laboratory of Bacteriology Research (LBR, Ghent University, Belgium). Shipment was performed using shipping boxes filled with dry ice (− 78.5 °C). Once at the LBR the Eppendorf tubes with DNA-extracts were transferred back to − 80 °C until molecular analysis was performed. No thawing and freezing occurred after freezing the DNA extract until the point of molecular testing in the laboratory in Belgium.

### Quantitative PCR

Most quantitative PCRs were performed using a highly sensitive and specific TaqMan® Array Card (TAC), developed at AZ Sint-Jan Brugge-Oostende, Belgium. The array card was used for detecting *Chlamydia trachomatis* (including Lymphogranuloma venereum (LGV) serovars L1-L2-L3), *Neisseria gonorrhoeae, Haemophilus ducreyi*, *Mycoplasma genitalium* (including *M. genitalium* macrolide resistance-mediating mutations A2058G, A2059G, A2058T, A2058C in region V of the 23S rRNA gene), *M. hominis*, *Ureaplasma parvum, U. urealyticum*, *Treponema pallidum*, Herpes Simplex virus-1/− 2 (HSV 1/2), adenoviruses, Cytomegalovirus (CMV), and *T. vaginalis*. The assay has multiple genetic targets per pathogen in order to maximize both specificity and sensitivity [9]. Samples were determined to be positive for a particular species on the TaqMan® Array Card (TAC), only in case the assay was positive for two independent PCR targets of that species. Sample quality was assessed by amplification of human DNA, to evaluate the quantity of epithelial cells recovered by the swab.

Further individual qPCRs were performed at the LBR for *Candida albicans* [10]*, Escherichia coli* [11] and *Streptococcus agalactiae* [11, 12]. The LightCycler 480 platform and the LightCycler 1480 Software Version 1.5 (Roche) were used for the amplification, detection and quantification. Each qPCR was performed in a final volume of 10 μl of which 2 μl of DNA extract or 2 μl of a negative control (HPLC water) or 2 μl of a positive control. All the specific primers and probes were synthesized by Eurogentec, Liège, Belgium. Specific qPCR details are provided in Additional file 1: Table S2.

The procedures as described by Cools et al. [10] were adopted for the construction of qPCR standard curves. Briefly, DNA was extracted from overnight cultures of *C. albicans* ATCC 90028 grown on Sabouraud agar (Becton Dickinson, Erembodegem, Belgium) and of *E. coli* ATCC 25922 or *S. agalactiae* LMG 14694^T^ grown on TSA + 5% sheep blood (Becton Dickinson). All colonies were collected from the plate and re-suspended in 1 ml of saline. The manufacturer’s instructions of the High Pure PCR Template Preparation Kit (Roche Applied Science, Basel, Switzerland) were followed to extract DNA from this suspension. DNA-concentration was determined by means of Nanodrop and the number of genomes was calculated. A tenfold dilution series in HPLC-grade water was made to establish for each dilution the number of Cqs needed to pass the detection threshold. Using these data, a regression curve was constructed.

### PCR for *T. vaginalis* and for Candidatus *Mycoplasma girerdii* (Ca. *M. girerdii*)

PCRs for *T. vaginalis* targeting the *actin* gene, using outer primers, previously used in a nested PCR [13] and yielding a fragment of 1100 bp and for Ca. *M. girerdii*, yielding a fragment of 594 bp [14], were carried out on the ABI Veriti thermocycler platform (ThermoFisher Scientific, Waltham, Massachusetts). The primers were synthesized by Eurogentec, Liège, Belgium. Amplified fragments were visualized under UV light after agarose gel electrophoresis and EtBr staining.

Details of these species-specific PCRs are summarized in Additional file 1: Table S2.

### Sequencing

Sequencing of PCR amplicons was carried out by GATC Biotech (Constance, Germany) to confirm specificity of the PCR products. Sequencing was done using the forward PCR primers (Additional file 1: Table S2). Sequences were cleaned using Chromas Lite version 2.1 (Technelysium, Brisbane, Australia). BLAST was performed on the sequences to confirm the identity.

### Data analysis

Epidemiological data were analyzed using StataCorp. 2013. Stata Statistical Software: Release 13 (College Station, TX: StataCorp LP). Prevalence of urogenital pathogens were computed with 95% confidence intervals (CIs). Associations between *T. vaginalis* positivity and socio-demographic, hygienic and behavioral characteristics were calculated using the χ2 test. To build a multivariate model of urogenital species associated with *T. vaginalis*, we first carried out univariate regression analysis. For computation of odds ratios (ORs), we replaced all zero values in cells by the value ‘0.5’, as suggested by Deeks & Higgins [13].

Species that were significantly associated with *T. vaginalis* in univariate regression analysis *P*-value ≤0.1 were selected for multivariate logistic regression analysis. Associations in the final multivariate model were expressed as adjusted odds ratios (AORs) with *p*-values ≤0.05 considered significant. We further assessed whether pathogens, significantly associated with *T. vaginalis* infection, had an implication on the clinical presentation.

## Results

A total of 23 out of 350 samples (6.5%) were positive by InPouch culture for *Trichomonas vaginalis,* of which eight (34.8%) were initially positive on direct microscopy. The *T. vaginalis*-specific PCR [13] detected one additional case of *T. vaginalis* from a sample that was negative by InPouch culture but positive by the TAC assay, which found four more positive samples. In summary, sensitivity of direct microscopy, of *T. vaginalis* InPouch culture and of *T. vaginalis*-specific PCR were respectively 28.6, 82.1 and 85.7%, when compared to the TAC assay.

Distribution of age, religion, education level, marital status, parity, gestational age and number of lifetime sex partners were similar among cases and controls (Table 1). HIV and BV status was different between the two groups by our case-control study design (Table 2), but the resulting overall difference was minimal, i.e. 0 HIV and 0 BV cases in the control group compared to 3 HIV-positives (10.7%) and 4 BV-positives (14.3%) in the *Trichomonas* positive group. Moreover, we could show that these differences had no influence on the species associated with *T. vaginalis* (Additional file 1: Tables S3 and S4). Additionally, occupation was also significantly different (Table 1).

**Table 1:** Characteristics of *Trichomonas vaginalis* qPCR positive women (cases) and *T. vaginalis* qPCR negative women (controls)

Characteristic	Cases (%) *N* = 28	Controls (%) *N* = 51	χ2 x *P*-value
Age group (Years)			
18–24	42.9	31.4	0.307
≥ 25	57.1	68.6	
Religion			
Christian	64.3	68.6	0.383
Muslim	10.7	17.7	
Other/None	25.0	13.7	
Education			
None	21.4	23.5	0.946
Primary	60.7	56.9	
Secondary/Tertiary	17.9	19.6	
Employment status			
Employed/self-employed	50.0	72.6	**0.045**
Unemployed	50.0	27.5	
Parity			
0	25.0	35.3	0.234
1–2	46.4	25.4	
3+	28.6	37.3	
Gestational age (weeks)			
14–27	57.1	58.8	0.885
≥ 28	42.9	41.2	
Number of lifetime sex partners			
1	82.1	90.2	0.303
3+	21.7	9.8	

In bold: significantly associated, i.e., *P* ≤ 0.05

**Table 2:** Prevalence and co-occurrence of urogenital species among 28 *Trichomonas vaginalis-*positive and 51 *T. vaginalis-*negative women

Species	Overall prevalence (*N* = 79) (95% CI)	%TV+/%TV-	Univariate analysis		Multivariate analysis	
			COR (95% CI)	*P*-value	AOR (95% CI)	*P*-value
*Candida albicans*	24.1 (15.1–35.0)	32.1/19.6	1.9 (0.7–5.6)	0.216	–	–
*Chlamydia trachomatis*	13.9 (7.2–23.5)	21.4/9.8	2.5 (0.7–9.1)	0.163	–	–
*Escherichia coli*	27.8 (18.3–39.1)	35.7/23.5	1.8 (0.7–4.9)	0.251	–	–
*Mycoplasma genitalium*	6.3(2.1–14.2)	17.9/0.0	5.0 (0.3–94.3)^*^	**0.002**		
Ca. *Mycoplasma girerdii*	7.6 (2.8–15.8)	21.4/0.0	4.3 (0.2–78.4)^*^	**0.001**		
*Mycoplasma hominis*	41.8 (30.8–53.4)	71.4/25.5	7.3 (2.6–20.5)	**< 0.001**	6.8 (2.3–19.8)	**< 0.001**
*Streptococcus agalactiae*	11.4 (5.3–20.5)	7.1/13.7	0.5 (0.1–2.5)	0.386	–	–
*Ureaplasma parvum*	74.7 (63.6–83.8)	78.9/72.6	1.4 (0.5–4.1)	0.557	–	–
*Ureaplasma urealyticum*	48.1 (36.7–59.6)	60.7/41.2	2.2 (0.9–5.7)	0.099	1.3 (0.4–3.8)	0.624
Cytomegalovirus	1.3 (0–6.9)	0.0/2.0	1.7 (6.6–4210.4)^*^	0.456		
HIV	3.8 (0.7–10.7)	10.7/0.0	7.9 (0.4–158.5)^*^	**0.017**		
HSV 1, HSV 2	2.5 (0.3–8.8)	7.1/0.0	11.1 (0.5–238.6)^*^	0.053		
Bacterial vaginosis	5.1 (1.4–12.5)	14.3/0.0	6.2 (0.3–118.4)^*^	**0.006**		

*TV Trichomonas vaginalis*, *HIV* human immunodeficiency virus, *HSV1* herpes simplex virus type 1; herpes simplex virus type 2, *COR* crude odds ratio, *AOR* adjusted odds ratio

in bold: significantly associated, i.e., *P* ≤ 0.05^*^ Separate computation not included in multivariable model

### Prevalence of urogenital species

The prevalence of the co-infecting urogenital species is indicated in Table 2. Adenovirus, *Haemophilus ducreyi, Neisseria gonorrhoeae* and *Treponema pallidum* were not detected and are not reported in Table 1. *Ureaplasma parvum* was the most prevalent at 74.7% (95% Confidence interval (CI): 63.6–83.8), followed by *U. urealyticum* at 48.1% (CI: 36.7–59.6).

### Univariate and multivariate association analysis

Although, *M. genitalium* and Ca. *M. girerdii*, had generally a low prevalences of respectively 6.3 and 7.6%, the two were exclusively detected in women with *T. vaginalis* (Chi-square test: χ^2^ = 9.7, df = 1, *P* <  0.002 and χ^2^ = 11.8, df = 1, *P* <  0.001, respectively). Both *M. genitalium* and Ca. *M. girerdii* were significantly associated with *T. vaginalis* on univariate analysis but not on multivariable analysis (Table 2). None of the samples for which *M. genitalium* could be detected showed macrolide resistance-associated mutations. In a univariate regression analysis, *M. hominis* and *U. urealyticum* were significantly associated with *T. vaginalis* (crude odds ratio (COR) = 7.3; 95% CI: 2.6–20.5 and COR = 2.2; 95% CI: 0.9–5.7, respectively). We detected *M. hominis* from the vaginal DNA extracts of approximately 70% of women with *T. vaginalis. M. hominis* was also independently associated with *T. vaginalis* in a multivariate regression analysis (adjusted odds ratio (AOR) = 6.8; 95% CI: 2.3–19.8).

Tables 3, 4 and 5 compare clinical signs and symptoms among *T. vaginalis*-infected women co-infected or not with *M. hominis* (Table 3), or with Ca *M. girerdii* (Table 4) or with *M. genitalium* (Table 5).

**Table 3:** Clinical signs/symptoms among women co-infected with *Trichomonas vaginalis* and *Mycoplasma hominis* versus *T. vaginalis* only

Clinical sign or symptom	TV with MH *N* = 20 (%)	TV without MH *N* = 8 (%)	χ2 *P*-value
Dyspareunia	40.0	37.5	0.903
Dysuria	30.0	37.5	0.701
Foul smelling vaginal odor	30.0	12.5	0.334
Genital ulcers	15.0	12.5	0.864
Genital warts	10.0	0.0	0.353
Lower abdominal pain	40.0	25.0	0.454
Vaginal discharge	75.0	62.5	0.508
Vaginal itching	30.0	25.0	0.791

*MH Mycoplasma hominis*, *TV Trichomonas vaginalis*

**Table 4:** Clinical signs/symptoms among women co-infected with *Trichomonas vaginalis* and Ca *Mycoplasma girerdii* versus *T. vaginalis* only

Clinical sign or symptom	TV with Ca MG *N* = 6 (%)	TV without Ca MG *N* = 22 (%)	χ2 *P*-value
Dyspareunia	50.0	36.4	0.544
Dysuria	50.0	27.3	0.291
Foul smelling vaginal odor	33.3	22.7	0.595
Genital ulcers	16.7	13.6	0.851
Genital warts	0.0	9.1	0.443
Lower abdominal pain	50.0	32.8	0.410
Vaginal discharge	100.0	63.6	0.081
Vaginal itching	66.7	18.2	**0.020**

*Ca MG* Candidatus *Mycoplasma girerdii*, *TV Trichomonas vaginalis*; in bold: significantly associated

**Table 5:** Clinical signs/symptoms among women co-infected with *Trichomonas vaginalis* and *Mycoplasma genitalium* versus *T. vaginalis* only

Clinical sign or symptom	TV with MG *N* = 5 (%)	TV without MG *N* = 23 (%)	(χ2).P-value
Dyspareunia	20.0	43.5	0.330
Dysuria	20.0	34.8	0.521
Foul smelling vaginal odor	40.0	21.7	0.393
Genital ulcers	0.0	17.4	0.314
Genital warts	0.0	8.7	0.494
Lower abdominal pain	40.0	34.8	0.825
Vaginal discharge	80.0	69.6	0.640
Vaginal itching	0.0	34.8	0.119

*MG Mycoplasma genitalium*, *TV Trichomonas vaginalis*

Women co-infected with *T. vaginalis* and Ca. *M. girerdii* were more likely to report vaginal itching compared to *T. vaginalis*-positive women not co-infected with Ca. *M. girerdii* (66.8% vs. 18.2% (*p* = 0.020)). There was no difference in clinical presentation of *T. vaginalis*-infected pregnant women co-infected with *M. hominis* or with *M. genitalium,* compared to those not co-infected with these species.

Of the five participants that had *M. genitalium* and the six that had Ca *M. girerdii*, only one participant had a co-infection with *M. genitalium* and Ca *M. girerdii*. However, *M. hominis* was always present in vaginal samples from which *M. genitalium* and Ca *M. girerdii* were detected. Due to the detection of CMV, HSV 1/ 2, *M. genitalium* and Ca. *M. girerdii* exclusively in cases or controls, the species were excluded from the regression model.

## Discussion

Our results indicate that women with *Trichomonas vaginalis* (*n* = 28) have a high rate (71.4%) of co-infection with *Mycoplasma hominis* compared to only 25.5% of 51 women not infected with *T. vaginalis.* Comparable rates of co-infection have been reported by Becker et al. [15], i.e., 56.7% in Brazil and by Xiao et al. [16], i.e., 50.0% in China. Rappelli et al. [17] reported much higher rates (94.3%) of co-infection among Italian, Mozambican, and Angolan women. *M. hominis* has been shown to have a symbiotic relationship with *T. vaginalis.* Owing to its small genome, this bacterial species is strongly dependent on host cell metabolism. *M. hominis* has the ability to enter trichomonad cells by endocytosis and to multiply in coordination with the protozoan [5]. We could not establish differences in clinical presentation of women co-infected with both *T. vaginalis* and *M. hominis* as compared to those infected with only *T. vaginalis*, in agreement with previous data [18]. As such, at present, co-infection of *T. vaginalis* with *M. hominis* seems to be of limited clinical relevance, also because antibiotic treatment of the former will probably consecutively diminish the presence of the latter.

The pathogenic potential of *M. genitalium* among pregnant women in Kenya has not been extensively investigated probably because its prevalence is overshadowed by a higher prevalence of other STIs, as was the case in this study. Our results indicate that in our population *M. genitalium* was strongly associated with *T. vaginalis* (*p* = 0.002). Given the presence of *M. genitalium* exclusively in women with *T. vaginalis* infection, screening and treatment of women for *T. vaginalis* might also at once reduce the prevalence of *M. genitalium.* Although macrolide resistance associated mutations among *M. genitalium* strains are on the rise, as was recently shown among female sex workers in Belgium [19], macrolide resistance-associated mutations could not be detected in any of the five samples positive for *M. genitalium* and therefore our results, although based on a very small sample, suggest that macrolides can still be used for treatment of *M. genitalium* in this population in Kilifi, Kenya.

To our knowledge, this is the first report of Ca. *M. girerdii* in Africa. In agreement with the two initial reports on the prevalence of Ca. *M. girerdii* [20, 21], we found it to be strongly associated with *T. vaginalis* (*p* **=** 0.001**)**. Fettweis et al. [20], using a pyrosequencing approach, were the first to detect Ca. *M. girerdii* DNA in the vaginal swabs of a few women not infected with *T. vaginalis,* as assessed with qPCR, although in our study, Ca. *M. girerdii* was found only in *T. vaginalis* positive women. A recent report by Costello et al. [22] is in support of the close association of Ca. *M. girerdii* and *T. vaginalis* as they recovered Ca. *M. girerdii* and *T. vaginalis* genomes from the saliva of a premature infant.

Our data suggest that *T. vaginalis*-positive women, co-infected with Ca. *M. girerdii,* were more likely to report vaginal itching compared to *T. vaginalis* mono-infected women. Future studies should elucidate the nature of the interaction of these two pathogens and the effect that co-infection may have on clinical presentation.

*U. parvum* and *U. urealyticum* are commonly isolated from the vaginal microbiome of asymptomatic pregnant women [23], as was the case in our study. Although detection of *U. parvum* has been associated with preterm birth [24], opinions differ with regard to the need to screen and treat *Ureaplasma* spp. infection during pregnancy, since its presence often represents colonization rather than infection [25]. Our data did not show any association between *T. vaginalis* and either *U. parvum* or *U. urealyticum*.

*C. trachomatis* was highly prevalent (13.9%) in our study. All isolates were non-LGV strains, but were not associated with *T. vaginalis* infection. Our results on urogenital carriage of *Candida*, *E. coli*, and GBS indicate that the three were not associated with *T. vaginalis* infection, either. While the prevalence of *Candida* in our study was higher than that reported in a cross-sectional study by Cools et al. [11] among women in Kenya, Rwanda and South-Africa, our prevalence for *E. coli* and GBS is comparable to what they reported.

Our study had some limitations. First, it only included a relatively small sample size of pregnant women limiting the precision of our prevalence estimates. Furthermore, only BV negative samples were included in the control arm, which may represent a bias on the interpretation of the results. Among the 28 *T. vaginalis*-positive women, only four were positive for BV and excluding them in the analysis does not affect the results (Additional file 1: Table S3). It should be noted that our *T. vaginalis*/BV co-infection rate of 14% was comparable to that observed in a recent study, i.e. 17.5% among HIV + women [26]. Finally, no internal control was added during the DNA extraction process, so inefficient genome extraction or (partial) PCR inhibition could not be documented. However, sample adequacy was evaluated by detecting a minimal level of human DNA present in the sample, which was the case for all samples, moreover none of the samples that were culture-positive for *T. vaginalis* were missed by PCR.

## Conclusion

We observed notable prevalence of urogenital micro-organisms, pathogens and colonizing germs among pregnant women which emphasizes the need for laboratory testing and treatment to avoid unfavorable pregnancy outcomes. We confirm *M. hominis* as a correlate of *T. vaginalis* in our population, but the most salient finding was the exclusive association of both *M. genitalium* and Ca. *M. girerdii* with *T. vaginalis*. The latter finding ought to be further addressed using a larger sample size.

## Additional file

Additional file 1: **Table S1.** Socio-demographic and behavioral characteristics of women selected as controls and women not selected as controls, attending prenatal care at Kilifi County Hospital, Kenya. **Table S2.** Specific PCR details. **Table S3.** Prevalence of urogenital species among *Trichomonas vaginalis* qPCR positive women (24 cases) and *T. vaginalis* qPCR negative women (51 controls) and Univariate and Multivariate analysis of the presence of species among cases vs controls among 75 women attending prenatal care at Kilifi County Hospital, Kenya. **Table S4.** Prevalence of urogenital species among *Trichomonas vaginalis* qPCR positive women (22 cases) and *T. vaginalis* qPCR negative women (51 controls) and Univariate and Multivariate analysis of the presence of species among cases vs controls among 73 women attending prenatal care at Kilifi County Hospital, Kenya. (DOCX 22 kb)

## Abbreviations

BV: Bacterial vaginosis
Ca. *M. girerdii*: Candidatus *Mycoplasma girerdii*
*M. genitalium*: *Mycoplasma genitalium*
M. *hominis*: *Mycoplasma hominis*
PCR: Polymerase chain reaction
STIs: Sexually transmitted infections
*T. vaginalis*: *Trichomonas vaginalis*
TAC: TaqMan® Array Card
*U. parvum*: *Ureaplasma parvum*
*U. urealyticum*: *Ureaplsama urealyticum*
