# Supplementary material for: Urogenital pathogens, associated with Trichomonas vaginalis, among pregnant women in Kilifi, Kenya: a nested case-control study
Source: BMC Infect Dis. 2018 Nov 6;18:549. doi: 10.1186/s12879-018-3455-4 (PMC6219184; doi:10.1186/s12879-018-3455-4)
Supplement: Supplementary file 1 — Table S1. Socio-demographic and behavioral characteristics of women selected as controls and women not selected as controls, attending prenatal care at Kilifi County Hospital, Kenya. Table S2. Specific PCR details. Table S3. Prevalence of urogenital species among Trichomonas vaginalis qPCR positive women (24 cases) and T. vaginalis qPCR negative women (51 controls) and Univariate and Multivariate analysis of the presence of species among cases vs controls among 75 women attending prenatal care at Kilifi County Hospital, Kenya. Table S4. Prevalence of urogenital species among Trichomonas vaginalis qPCR positive women (22 cases) and T. vaginalis qPCR negative women (51 controls) and Univariate and Multivariate analysis of the presence of species among cases vs controls among 73 women attending prenatal care at Kilifi County Hospital, Kenya. (DOCX 22 kb) [file 12879_2018_3455_MOESM1_ESM.docx]

**Table S1. Socio-demographic and behavioral characteristics of women selected as controls and women not selected as controls, attending prenatal care at Kilifi County Hospital, Kenya.**

| **Characteristic** | **Not selected as controls (%)**  **N = 270*** | **Selected as controls (%)**  **N = 51** | **χ2**  **P-value** |
| --- | --- | --- | --- |
| **Age group (Years)** |  | | |
| 18-24 | 38.5 | 31.4 | 0.333 |
| ≥ 25 | 61.5 | 68.6 |  |
| **Religion** |  | | |
| Christian | 72.2 | 68.6 | 0.872 |
| Muslim | 15.6 | 17.7 |  |
| Other/None | 12.2 | 13.7 |  |
| **Education** |  | | |
| None | 16.7 | 23.5 | 0.387 |
| Primary | 57.0 | 56.9 |  |
| Secondary/Tertiary | 26.3 | 19.6 |  |
| **Employment status** |  | | |
| Employed/self-employed | 55.2 | 72.6 | **0.021** |
| Unemployed | 44.8 | 27.5 |  |
| **Parity** |  | | |
| 0 | 25.6 | 35.3 | 0.207 |
| 1-2 | 39.3 | 25.4 |  |
| 3+ | 35.2 | 37.3 |  |
| **Gestational age (weeks)** |  | | |
| 14-27 | 63.7 | 58.8 | 0.511 |
| ≥ 28 | 36.3 | 41.2 |  |
| **Number of lifetime sex partners** |  | | |
| 1 | 88.9 | 90.2 | 0.784 |
| 3+ | 11.1 | 9.8 |  |

**Legend**

In bold: significantly associated, *i.e.*, P ≤ 0.05

* Socio-demographic and behavioral characteristics for one participant were missing and thus 270 and not 271.

**Table S2. Specific PCR details**

| **Target organism**  **[reference]** | **Composition and primer reference (final volumes: 10 µl)*** | **Thermocycler conditions** |
| --- | --- | --- |
| *Candida albicans*  [9] | LightCycler 4801SYBR Green I Master (Roche): 5 µl  Forward primer: 0.3 μM CA_FW:  CAACGAACTGAACTGGCAGA  Reverse primer: 0.3 µM CA_RV:  CATTACGCTGCGATGGAT | 50 °C for 2 min  95 °C for 10 min  40 x (95 °C for 15 s and 60 °C for 1 min) |
| *Escherichia coli*  [10] | LightCycler 4801 SYBR Green I Master (Roche): 5 μl  Forward primer: 0.3 μM Ecoli_FW:  CAACGAACTGAACTGGCAGA  Reverse primer: 0.3 μM Ecoli_RV:  CATTACGCTGCGATGGAT  Probe: 0.3 μM  TATCCCGCCGGGAATGGTGA | 50 °C for 2 min  95 °C for 10 min  40 x (95 °C for 15 s and 60 °C for 1 min) |
| *Streptococcus agalactiae*  [11] | LightCycler 4801SYBR Green I Master (Roche): 5 μl  Forward primer: 0.5 μM Sip1:  ATCCTGAGACAACACTGACA  Reverse primer: 0.5 μM Sip2  TTGCTGGTGTTTCTATTTTCA  Probe: 0.3 μM  6-FAM–ATCAG AAGAGTCATACTGCCACTTC–TAMRA | 95 °C for 5 min  40 x (95 °C for 10 s, 58 °C for 15 s and 72 °C for 20 s) |
| *Trichomonas vaginalis*  [13] | Faststart PCR master (Roche): 10 μl  Forward primer: 0.2 μM Tv8S  TCT GGA ATG GCT GAA GAA GAC G  Reverse primer: 0.2 μM Tv9R  CAG GGT ACA TCG TAT TGG TC | 95 °C for 5 min  40 x (95 °C for 30 s, 58 °C for 30 s and 72 °C for 3min)  72°C for 7 min |
| Candidatus *Mycoplasma girerdii*  [14] | Faststart PCR master (Roche): 10 μl  Forward primer: 0.2 μM M1_Contig1-3_F:  TGCATATCCATCTAATGCAACC  Reverse primer: 0.2 μM M1_Contig1-3_R:  AGGGACTGTATTTTATCGCAATGG | 95 °C for 5 min,  40 × (95 °C for 30 s, 50 °C for 30 s and 72 °C for 45 s)  72°C for 7 min |

**Legend**

* Candidatus *Mycoplasma girerdii* and *Trichomonas vaginalis* PCRs were carried out in a total volume of 20 μl.

**Table S3. Prevalence of urogenital species among *Trichomonas vaginalis* qPCR positive women (24 cases) and *T. vaginalis* qPCR negative women (51 controls) and Univariate and Multivariate analysis of the presence of species among cases vs controls among 75 women attending prenatal care at Kilifi County Hospital, Kenya. (Four women with BV are excluded)**

| **Species** | **Overall prevalence**  **(N = 75) (95% CI)** | **N = 75**  **(24/51)**  **(%TV+/%TV-)** | **Univariate analysis** | | **Multivariate analysis** | |
| --- | --- | --- | --- | --- | --- | --- |
|  |  |  | **COR (95% CI)** | **P-value** | **AOR (95% CI)** | **P-value** |
| *Candida albicans* | 24.0 (14.9-35.1) | 33.3/19.6 | 2.1 (0.7-6.1) | 0.199 | - | - |
| *Chlamydia trachomatis* | 13.3 (6.5-23.2) | 20.8/9.8 | 2.4 (0.6-9.3) | 0.199 | - | - |
| *Escherichia coli* | 26.7 (17.1-38.1) | 33.3/23.5 | 1.6 (0.6-4.7) | 0.373 | - | - |
| *Mycoplasma genitalium* | 5.3 (1.5-13.1) | 16.7/0.0 | 5.3 (0.3-102.1)* | **0.003** |  |  |
| Ca. *Mycoplasma girerdii* | 8.0 (3.0-16.6) | 25.0/0.0 | 3.7 (0.2-67.6)* | **<0.001** |  |  |
| *Mycoplasma hominis* | 38.7 (27.6-50.6) | 66.7/25.5 | 5.8 (2.0-16.8)* | **0.001** | 5.5 (1.8-16.4) | **0.002** |
| *Streptococcus agalactiae* | 10.6 (4.7-19.9) | 4.2/13.7 | 0.3 (0.0-2.4) | 0.386 | - | - |
| *Ureaplasma parvum* | 74.7 (63.3-84.0) | 79.2/72.6 | 1.4 (0.5-4.6) | 0.540 | - | - |
| *Ureaplasma urealyticum* | 46.7 (35.1-58.6) | 58.3/41.2 | 2.0 (0.7-5.4) | 0.168 | 1.3 (0.4-3.8) | 0.678 |
| Cytomegalovirus | 1.3 (0-7.2) | 0.0/2.0 | 1.4 (5.6-3631.8)* | 0.490 |  |  |
| HIV | 2.7 (0.3-9.3) | 8.3/0.0 | 9.5 (0.4-205.8)* | **0.037** |  |  |
| HSV 1, HSV 2 | 1.3 (0.0-7.2) | 4.2/0.0 | 15.9 (0.6-403.5)* | 0.142 |  |  |

**Legend:** TV: *Trichomonas vaginalis*; HIV: human immunodeficiency virus; HSV1; HSV2: herpes simplex virus type 1 or type 2; COR: crude odds ratio; AOR: adjusted odds ratio; in bold: significantly associated, *i.e.*, P ≤ 0.05, * Separate computation not included in multivariate analysis.

**Table S4. Prevalence of urogenital species among *Trichomonas vaginalis* qPCR positive women (22 cases) and *T. vaginalis* qPCR negative women (51 controls) and Univariate and Multivariate analysis of the presence of species among cases vs controls among 73 women attending prenatal care at Kilifi County Hospital, Kenya. (Six women with either HIV or BV are excluded)**

| **Species** | **Overall prevalence**  **(N = 73) (95% CI)** | **N = 73**  **(22/51)**  **(%TV+/%TV-)** | **Univariate analysis** | | **Multivariate analysis** | |
| --- | --- | --- | --- | --- | --- | --- |
|  |  |  | **COR**  **(95% CI)** | **P-value** | **AOR**  **(95% CI)** | **P-value†** |
| *Candida albicans* | 24.7 (15.3-36.1) | 36.4/19.6 | 2.3(0.8-7.1) | 0.133 | **-** | **-** |
| *Chlamydia trachomatis* | 15.9 (7.9-27.3) | 22.7/9.8 | 2.7(0.7-10.5) | 0.151 | **-** | **-** |
| *Escherichia coli* | 26.0 (16.5-37.6) | 31.8/23.5 | 1.5 (0.5-4.6) | 0.460 | **-** | **-** |
| *Mycoplasma genitalium* | 5.5 (15.1-13.4) | 18.2/0.0 | 4.9 (0.3-94.0)* | **0.002** |  |  |
| Ca*. Mycoplasma girerdii* | 8.2 (3.1-17.0) | 27.3/0.0 | 3.4 (0.2-62.2)* | **<0.001** |  |  |
| *Mycoplasma hominis* | 38.4 (27.2-50.5) | 68.2/25.5 | 6.3 (2.1-18.7) | **0.001** | **5.5 (1.8-17.1)** | **0.003** |
| *Streptococcus agalactiae* | 11.0 (4.9-20.5) | 4.6/13.7 | 0.3 (0.0-2.6) | 0.273 | **-** | **-** |
| *Ureaplasma parvum* | 74.0 (62.4-83.5) | 77.3/72.6 | 1.2 (0.4-4.1) | 0.673 | **-** | **-** |
| *Ureaplasma urealyticum* | 47.9 (36.1-60.0) | 63.6/41.2 | 2.5 (0.9-7.0) | 0.082 | 1.6 (0.5.4.9) | 0.434 |
| Cytomegalovirus | 1.4 (0.0-7.4) | 0.0/2.0 | 1.3 (5.1-3342.5)* | 0.508 |  |  |
| HSV1, HSV2 | 1.4 (0.0-7.4) | 4.6/0.0 | 14.6 (0.6-371.4)* | 0.125 |  |  |

**Legend:** TV: *Trichomonas vaginalis*; HIV: human immunodeficiency virus; HSV1; HSV2: herpes simplex virus type 1 or type 2; COR: crude odds ratio; AOR: adjusted odds ratio; in bold: significantly associated, *i.e.*, P ≤ 0.05;* Separate computation not included in multivariate analysis.
